# Evaluation of intuitive trunk and non-intuitive leg sEMG control interfaces as command input for a 2-D Fitts’s law style task

**DOI:** 10.1371/journal.pone.0214645

**Published:** 2019-04-03

**Authors:** Stergios Verros, Koen Lucassen, Edsko E. G. Hekman, Arjen Bergsma, Gijsbertus J. Verkerke, Bart F. J. M. Koopman

**Affiliations:** 1 Department Biomechanical Engineering, University of Twente, Enschede, The Netherlands; 2 University of Groningen, University Medical Center Groningen, Groningen, The Netherlands; University of Illinois at Chicago, UNITED STATES

## Abstract

Duchenne muscular dystrophy (DMD) is a muscular condition that leads to muscle loss. Orthotic devices may present a solution for people with DMD to perform activities of daily living (ADL). One such device is the active trunk support but it needs a control interface to identify the user’s intention. Myoelectric control interfaces can be used to detect the user’s intention and consequently control an active trunk support. Current research on the control of orthotic devices that use surface electromyography (sEMG) signals as control inputs, focuses mainly on muscles that are directly linked to the movement being performed (intuitive control). However in some cases, it is hard to detect a proper sEMG signal (e.g., when there is significant amount of fat), which can result in poor control performance. A way to overcome this problem might be the introduction of other, non-intuitive forms of control. This paper presents an explorative study on the comparison and learning behavior of two different control interfaces, one using sEMG of trunk muscles (intuitive) and one using sEMG of leg muscles that can be potentially used for an active trunk support (non-intuitive). Six healthy subjects undertook a 2-D Fitts’s law style task. They were asked to steer a cursor into targets that were radially distributed symmetrically in five directions. The results show that the subjects were generally able to learn to control the tasks using either of the control interfaces and improve their performance over time. Comparison of both control interfaces demonstrated that the subjects were able to learn the leg control interface task faster than the trunk control interface task. Moreover, the performance on the diagonal-targets was significantly lower compared to the one directional-targets for both control interfaces. Overall, the results show that the subjects were able to control a non-intuitive control interface with high performance. Moreover, the results indicate that the non-intuitive control may be a viable solution for controlling an active trunk support.

## Introduction

Duchenne muscular dystrophy (DMD) is characterized by progressive skeletal muscle weakness and predominantly affects males with a prevalence of 1 per 6,000 [1]. Thanks to medication, the life expectancy of individuals with DMD has been increased and it is currently estimated to be over 30 years [2]. Loss of muscular activity leads to decrease in activities, participation and autonomy. Research has shown that Health Related Quality of Life (HRQoL) is significantly lower for people with muscular dystrophy when compared to healthy controls [3]. The increase of life expectancy with the decrease of muscle force consequently increases the dependency on caregivers.

Orthoses have the potential to assist people with DMD to perform activities of daily living. Some examples are arm orthoses that reduce gravity effects and assist the user in reaching tasks [4]. Those devices mainly provide passive support to overcome gravity, but research is being done on active arm orthoses for people with DMD [5]. In order to increase the reachable workspace, some level of trunk movement is important. Hence the Symbionics project was started with the aim to support trunk movements by orthotic devices. It was shown that that the movement of the trunk plays an important role in activities of daily living and that people with DMD can benefit from passive trunk support [6, 7]. However, with progressing DMD patients have less muscle capacitance and thus need additional assistance by an active support. Thus, part of the Symbionics project was dedicated to the development of an active trunk support. An extra benefit is an increase of the arm reach when an active trunk support is combined with an arm support [8]. To our knowledge, there is no active trunk assistive device yet.

To detect the user’s intention, surface electromyography (sEMG) can be used [9], [10]. It has been shown that adequate signals can be measured in persons with DMD and there is a straightforward relationship between the sEMG and the intended movement [11].

Using sEMG signals from muscles that are related to the movement of the supported anatomy is considered to be most intuitive to control an orthotic device. However, there may also be challenges to measure a sEMG on particular muscles. In the case of the trunk, one specific challenge is that respiratory muscles are located in the trunk as well and can easily disturb the control signal. Another is that muscles are often covered by fat, especially those in the abdomen, which consequently reduces the quality of the sEMG signal.

One way of overcoming those problems could be to record the sEMG from muscles that are not directly related to trunk movements (this is called non-intuitive in this article). The human brain has the ability to adapt to a certain level of non-intuitiveness [12]. It can form inverse models of space, optimize control strategies and learn new muscle synergies while completing physical tasks [13]. Furthermore, non-intuitive control interfaces have been used to drive a wheel chair [14]. The aim of this study was to evaluate the performance of an intuitive and a non-intuitive control interface for active trunk support. Regarding the non-intuitive control interface, we chose to record sEMGs from the tibialis anterior and gastrocnemious, muscles in the legs, because those muscles can be contracted easily while sitting and are usually covered by less fat tissue than the trunk muscles. While the performance of an intuitive and a non-intuitive interface that controls a 2-D cursor using arm muscles has been studied before, no known study has compared control interfaces based on sEMG signals from the legs and trunk [15].

To compare an intuitive control interface with a non-intuitive control interface, we designed and performed a 2-D experiment based on Fitts’s law under isometric conditions. The information that a motor task can convey is finite and this limitation of information is a consequence of the effort required for performing a movement both as rapidly and as accurately as possible [16]. This speed/accuracy trade-off is known as Fitts’s law and it holds for human motor tasks [17]. Moreover, it has been used for comparing different control interfaces [17].

The hypothesis is that the performance of the non-intuitive control interface is comparable to the intuitive one and thus can be used to control an active trunk support.

## Materials and methods

### Participants

Six healthy volunteers, without any prior experience of sEMG-based control, were recruited after obtaining informed consent. The research was explorative and thus did not fall within the scope of the Dutch Medical Research Involving Human Subjects Act. The experiment consisted of a single session and did not pose any risks or burden to the subjects. The CMO Regio Arnhem-Nijmegen committee under Prof. Dr. P.N.R. Dekhuijzen had decided that the ethical approval is waived. Registration number: 2018-4933.

### Test set-up and signal acquisition

**Fig 1:**
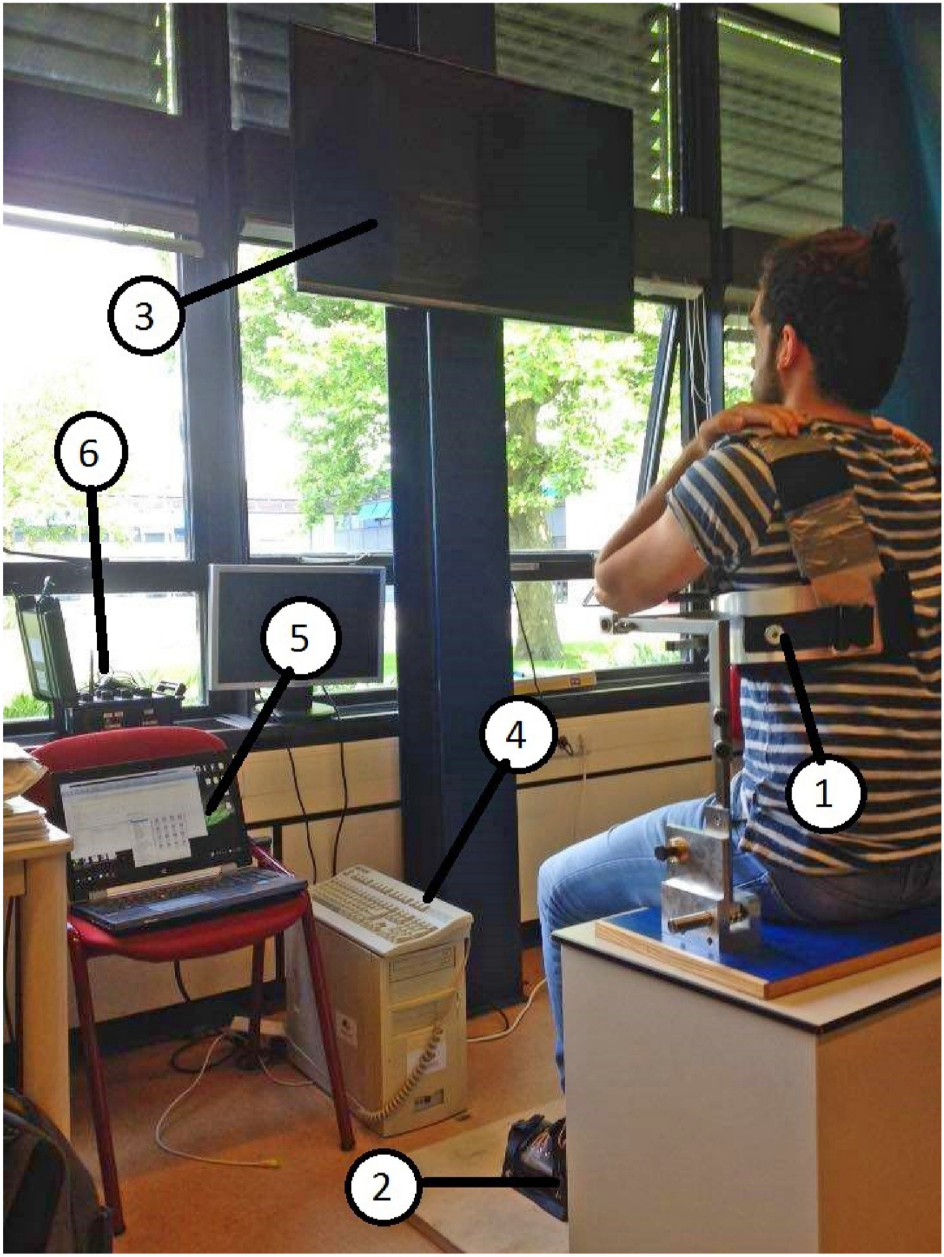
Test setup. In experiment A (intuitive control using trunk muscles), the subjects were seated on a chair while their trunk (1) and feet (2) were fixed with straps to the mechanical structure. In experiment B (non-intuitive control using leg muscles), only the subjects’ feet were fixed with straps (2). The data from the sEMG sensors were sent wireless to a base unit (6) that was connected to the xPC computer (4) which processed the data and sent the output to the PC (5). The screen (3) showed the cursor and the target to the subject.

The subjects sat on a mechanical frame that prevented any trunk or leg movement during the experiment (Fig 1). Due to the fact that the subjects were lifting their legs during experiment A we decided to fix them. On the other hand, subjects did not move their trunk during experiment B so we let the trunk free. The experimental setup included a personal computer (PC) with an external monitor to present visual feedback to the subjects and an xPC target computer with a data acquisition card ((PCI-6229; National Instruments Corp., USA, sampling at 1 KHz). The muscle activation signals were measured with wireless sEMG sensors (Trigno Delsys, USA).

### sEMG signal processing

In order to control a 2-D task, two pairs of antagonistic muscles were used for each of the two experiments. Each antagonistic pair of muscles controlled a 1-D movement. The cursor could also be controlled diagonally on the screen by a combination of the two antagonistic pairs. Regarding the trunk control interface, sEMG sensors were placed on the rector abdominis muscle (placement: 1/2 of the line between the belly bottom and groin area) and the erector spinae muscle (placement: 2 fingers lateral of the spine at L1 level) to control the vertical movement whereas the left and right oblique muscles (placement: 17 cm from belly button) were used to control horizontal movement. Regarding the leg control interface, sEMG sensors were placed on the left gastrocnemius muscle (placement: 1/3 of the line between the head of the fibula and the heel) and tibialis anterior muscle (placement: 1/3 of the line between the head of the fibula and the tip of the medial malleolus) for vertical movement whereas the right gastrocnemius muscle and tibialis anterior muscle were used to control horizontal movement.

The sEMG data were filtered with a high pass filter (3rd order Butterworth: cutoff 40 Hz), a rectifier function and subsequently, a low pass filter (3rd order Butterworth: cutoff 2 Hz). The normalized EMG signals, *sEMG*_*nor*,*k*_(*i*) were obtained using Eq (1).

$$\begin{array}{c} sEMG_{nor,k}\left(i\right)=(\frac{sEMG_{env,k}\left(i\right)-sEMG_{res,k}}{sEMG_{sub,k}}{)}^{1.5} \end{array}$$ (1)

where subscript k represents the abbreviations of the control muscles, *sEMG*_*env*,*k*_(*i*) denotes the processed sEMG signal at the i-th time step. *sEMG*_*sub*,*k*_ represents the submaximal contraction of the subject, *sEMG*_*res*,*k*_(*i*) represents the average signal amplitude during rest. The normalized signal was limited to a value of one to prevent velocities higher than the maximum velocity. The sub-maximum contraction value of each subject was determined by 2 seconds of sub-effort. The subjects were asked to contract their muscles at a level that did not need the maximum effort. The power function made the control of small movements easier.

After normalization, the muscles were mapped into the corresponding direction. The mapping function (*Q*) is a 2 × 4 matrix which maps the 4 × 1 vector *e* of the normalized sEMG amplitudes to a 2 × 1 vector *CursorVelocity* of control outputs. The first (last) two instances of the *e* vector are the cursor velocities of one pair of antagonistic muscles that steer the cursor in the horizontal (vertical) axis. The mapping function for this control can be seen in Eqs (2)–(4). *MaxVelocity* was set to the maximum fixed velocity of the cursor(400 pixels/s). The *sEMG*_*res*,*k*_(*i*) was also integrated in order to have a velocity mapping. Compared to position mapping, velocity mapping has the advantage to help the subjects to keep the cursor into the target without contracting their muscles. Each set of muscles only maps one of the two control axes required for the task (Table 1).

$$\begin{array}{c} CursorVelocity=Q*e*MaxVelocity \end{array}$$ (2)

$$\begin{array}{c} e=[\begin{array}{c} sEMG_{nor,1}\left(i\right)\\ sEMG_{nor,2}\left(i\right)\\ sEMG_{nor,3}\left(i\right)\\ sEMG_{nor,4}\left(i\right) \end{array}] \end{array}$$ (3)

$$\begin{array}{c} Q=[\begin{array}{cccc} -1 & 1 & 0 & 0\\ 0 & 0 & -1 & 1 \end{array}] \end{array}$$ (4)

**Table 1:** Muscle configuration.

Moving direction	Experiment A	Experiment B
*Right*	Right external oblique	Right tibialis anterior
*Left*	Left external oblique	Right gastrocnemious
*Up*	Rectus abdominis	Left gastrocnemious
*Down*	Eroctor spinae	Left tibialis anterior

This table shows the mapping of muscle activation with movements of the cursor on the screen

### Experimental procedure

**Fig 2:**
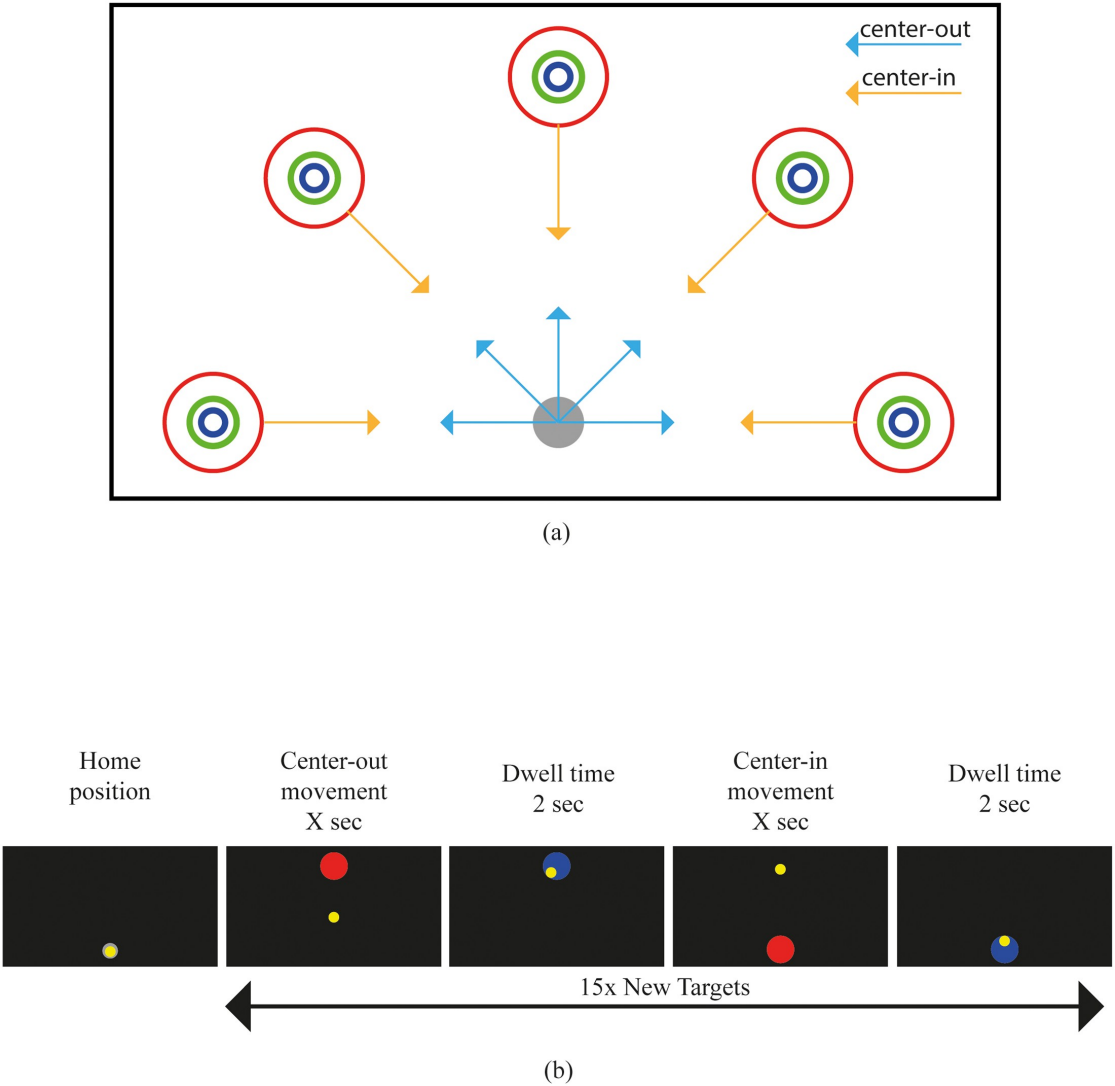
Experimental layout. (a) Targets configuration; (b) Trial sequence. Subjects performed 15-target center-out and center-in trials by controlling a cursor (yellow) from a starting area to one of the targets (red) shown on the screen.

Each set of muscles only maps one of the two control axes required for the task [17]. The experiment included two tasks: an intuitive controlled task (A) and a non-intuitive controlled task (B) and in the experiments the order was randomized per subject. In both tasks, the subjects were asked to steer the cursor into the target on the screen as quickly and accurately as possible by contracting different sets of muscles. According to Fitts’s law, the Index of Difficulty (ID) is defined by Eq (5), where D is the distance between the home position and the center of the target and W is the diameter of the target. In the current experiment, three different IDs were distinguished (2.3, 3.15 and 4.08). As the distance D was kept constant at 800 pixels, the differences in ID were only determined by the target widths of 200, 100 or 50 pixels. Targets were spaced radially from the initial position in 0°, 45°, 90°, 135°and 180°directions, as shown in (Fig 2A).

A trial consisted of two movements: one center-out movement (home to target) was followed by a center-in movement (target to home), both with the same ID. At the start, the cursor (yellow) was at the home position. The center-out movement started when a target (red) appeared on the screen in one of the 5 possible locations. The subject had to steer the cursor into the target zone and keep it there for a dwell time of 2-s. As soon as this had been achieved, a new target appeared at the home position. To give this center-in target the same ID as the center-out target, the diameter was adjusted based on the instantaneous location of the cursor at the end of the center-out movement. Finally, the experiment was subdivided in 5 blocks of 15 trials each, after which a break was inserted.

$$\begin{array}{c} ID=\log_{2}\left(\frac{D}{W}+1\right) \end{array}$$ (5)

### Performance metrics

The performance differences of experiments (A) and (B) were assessed by measuring Movement Time (MT), throughput (TP) and path efficiency (PE).

The Movement Time (MT) is the time that is needed for a successful trial without the 2-s target dwell time. To calculate the linear regression we followed Eq (6).

$$\begin{array}{c} MT=a\cdot ID+b \end{array}$$ (6)

Throughput (TP) is the information transfer rate and is a measure of the amount of information that the subject can process through a particular command source in relation to the task, in this case the cursor control [17]. The TP was computed using Eq (7).

$$\begin{array}{c} TP=\frac{ID}{MT} \end{array}$$ (7)

Path efficiency (PE) is a measure of the straightness of the cursor path to the target. Path efficiency is computed by Eq (8), where the linear distance between the points is divided by the actually travelled distance.

$$\begin{array}{c} PE=\frac{Linear\;Distance}{Actually\;Travelled\;Distance}\times 100\% \end{array}$$ (8)

Direction ratio shows the performance, TP and PE based on the direction. It is defined as the ratio of the average performance of each direction divided by the maximum performance.

Learning behavior is defined as the change of the performance of the throughput across the experimental blocks. A sigmoid function, Eq (9), was used to fit the data points.

$$\begin{array}{c} f\left(x\right)=\frac{1}{1+e^{-a(x-c)}}+b \end{array}$$ (9)

### Data analysis

The performance metrics described above were applied for the data analysis. The linearity between MT and ID was employed to check the consistency with Fitts’s law. MT, TP and PE were used to evaluate the performance of the two tasks considering only the last 3 blocks. The average of every experimental block was calculated for every metric and for the MT per ID as well. For the learning behavior, statistical analysis was performed between the TP of the last 2 blocks of the experiment. The Kolmogorov-Smirnov test showed that the data were not normally distributed. Thus, a non-parametric test, the Wilcoxon Signed Rank-test, was used with a level of significance at p<0.05.

## Results

Six subjects participated in the study. One subject did not complete experiment A (only three out of five blocks were completed). However, all the data of the six subjects were included in the analyses.

### Fitts’s law

**Fig 3:**
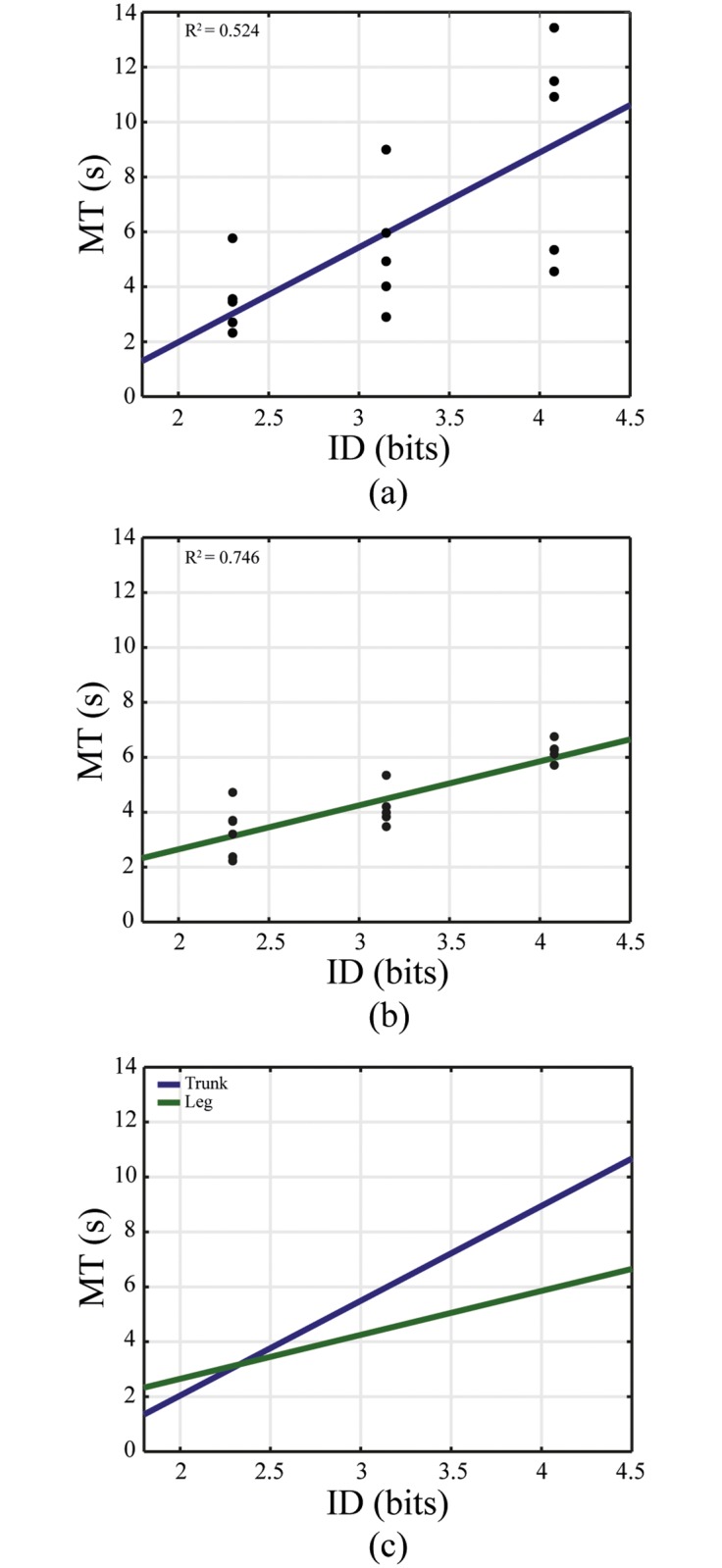
The linear regression of ID in bits versus the movement time (MT) in seconds. (a) Linear regression of the intuitive control interface, (b) of the non-intuitive control interface and (c) their comparison. The mean of each subject for every ID and control interface is indicated with a circle.

The plots of the regression analyses of MT versus ID are shown on Fig 3. The MT increases proportionally as the ID increases. The coefficient of determination for the non-intuitive control interface is higher (*R*^2^ = 0.746) than the intuitive control interface (*R*^2^ = 0.524).

### Movement time

**Fig 4:**
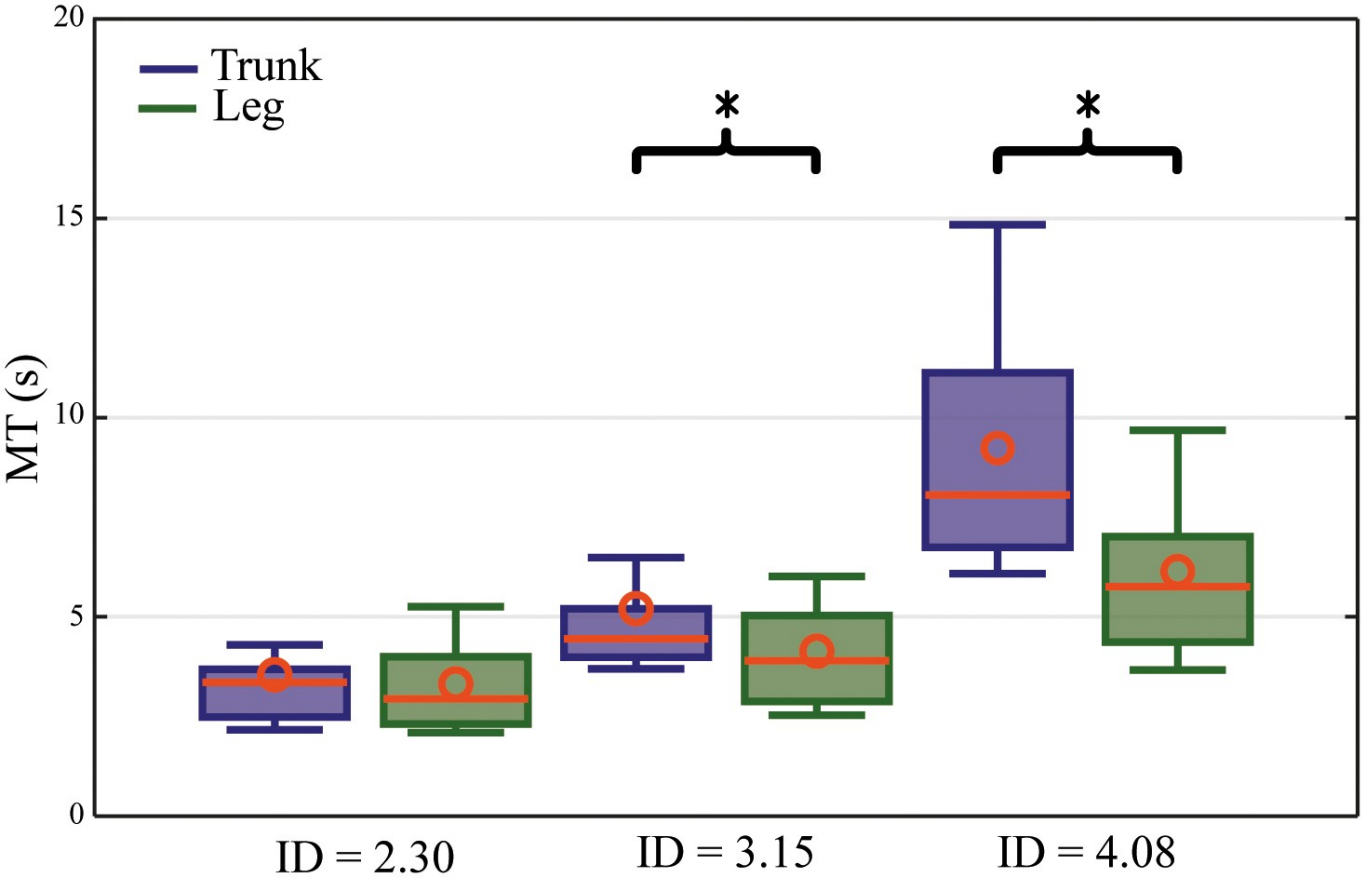
Boxplots of movement times. Boxplots of the movement times (MT) of the trunk and leg control interfaces, for each of the three IDs. * represents a statistical difference (p<0.05).

There was no significant difference in movement time between the two control interfaces for the lowest ID (2.3), whereas the 3.15 and 4.08 IDs demonstrated a significant difference in movement time between the control interfaces, with the non-intuitive control interface being faster Fig 4.

### Path efficiency and Throughput

**Fig 5:**
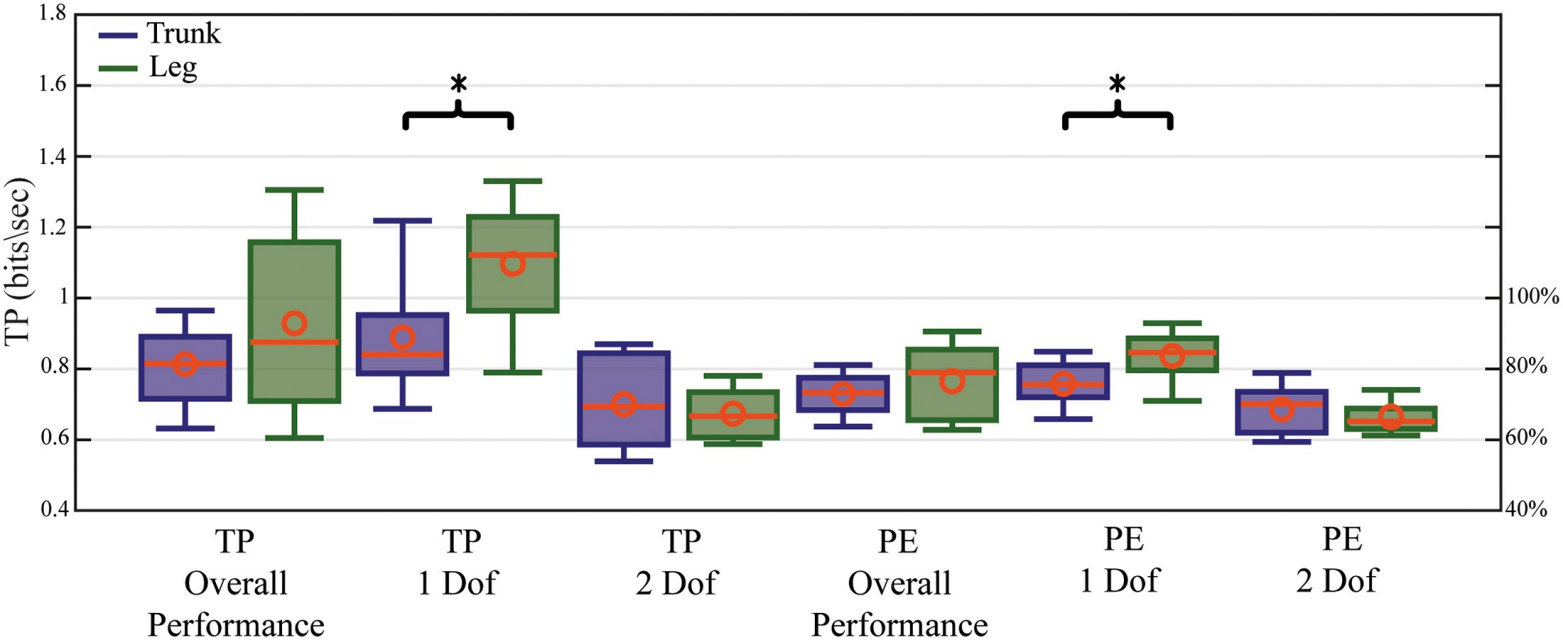
Path efficiency and Throughput comparison boxplots. Comparison of intuitive and non-intuitive controlled interfaces for throughput and path efficiency. Overall performance stands for all movements combined, 1-Dof for movements that only required one pair of antagonistic muscles, 2-Dof for movements that needed two pairs of antagonistic muscles.

The PE and TP performance metrics were analyzed for two conditions, one being independent of the target direction and the other being dependent on the target direction. The 1-Dof analyzed the movement whereby the subjects had to steer the cursor using only one pair of antagonistic muscles whereas the 2-Dof analyzed the subjects’ movement as they steered the cursor using two pairs of antagonistic muscles. Fig 5 shows that there is no significant difference between the two control interfaces in the overall performance of PE and TP. On the 1-Dof, the non-intuitive control interface showed greater performance and significance than the intuitive control interface, both in PE and TP. No significant difference was obtained on the 2-Dof.

**Fig 6:**
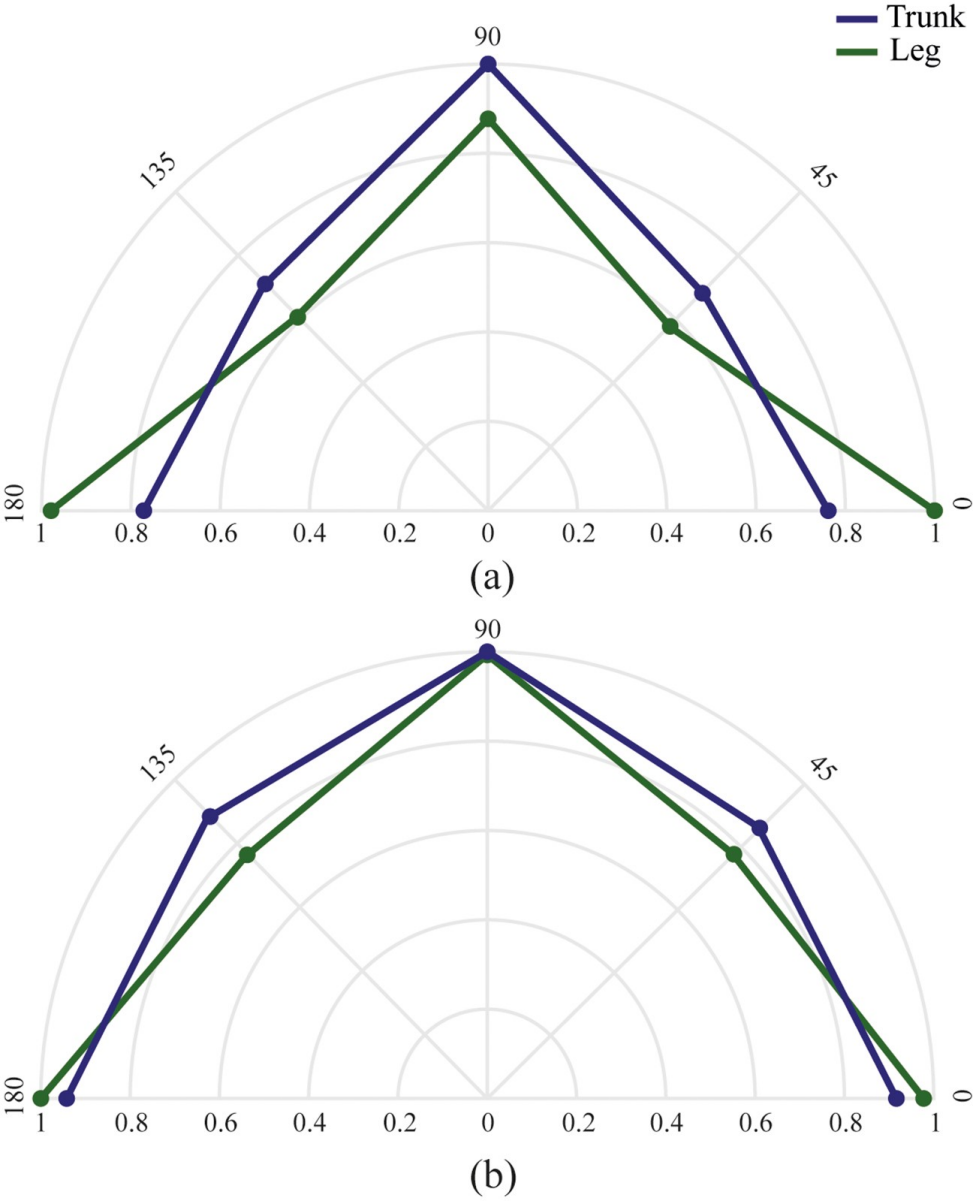
Path efficiency and Throughput path direction ratio. Normalized target-direction performance of the performance metrics Throughput (a) and Path efficiency (b); Blue indicates the non-intuitive control and red indicates the intuitive control interface.

The direction ratio shows the normalized performance from the highest PE and TP target direction Fig 6. Regarding the TP, the non-intuitive control interface performed the best in the left and right direction which was controlled by the right leg muscles. The lowest performance occurred in the diagonal direction where one muscle of each antagonistic pair was needed. The intuitive control interface showed the highest performance occurred with the backward and forward movements controlled by the rectus abdominis and the erector spinae. The diagonal targets performed the worst. Regarding the PE, both control interfaces performed highly in the single direction and less well in the diagonal directions.

### Learning behaviour

**Fig 7:**
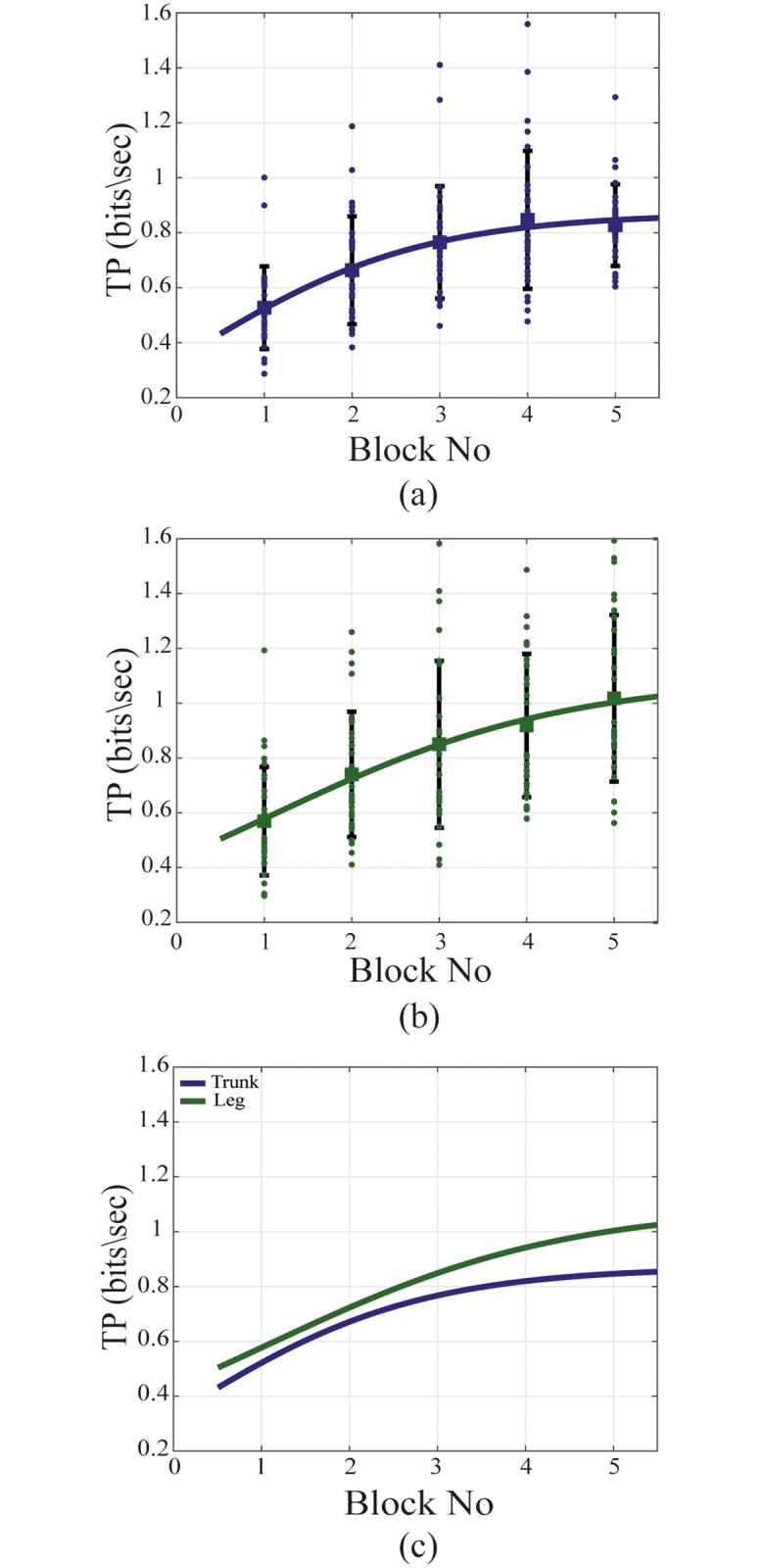
Learning curve. (a) shows the learning curve for the intuitive control interface, (b) for the non-intuitive control interface and (c) the comparison. The mean of every target (for the 6 subjects) is shown as a square shape and the error bar indicates one standard deviation from the mean in both directions.

The results of the statistical analysis between the last tasks indicate that both the leg and the trunk approached their steady performance state at the end of the experiment. There was no significant difference in throughput between the two last tasks for both control interfaces (for trunk p = 0.877, for leg p = 0.149). Fig 7 shows the Sigmoid fit together with the data points for both control interfaces individually and their comparison.

## Discussion

Control interfaces based on sEMG signals have the potential to control exoskeletons or orthotic devices. Intuitive control of an active trunk support based on sEMG from trunk muscles can be insufficient so non-intuitive sEMG control could be a valiable alternative solution. In this study, we compared the performance of two sEMG based control interfaces which could be potentially used for an active trunk support. The control interface with sEMG signals from trunk muscles was considered as intuitive whereas the control interface with sEMG signals from leg muscles was considered as non-intuitive.

### Fitts’s law

Fitts’s law seems to hold for both control interfaces although the R values are lower than in other studies. The low R values can be explained by the fact that a) a steady state of the learning behavior was not reached b) dwell time was used instead of tapping or pressing a button c) the muscles that were involved in the tasks are not made to perform the same fine movement tasks as the hand. The regression plot for the trunk shows a high spread in average MT per ID. When comparing the regression coefficients, it can be concluded that the non-intuitive control interface has a tighter relationship between ID and MT based on the coefficient of determination. A note needs to be made that the targets in the experiment only varied in size and that the distance was kept constant, thus only the target size effect of Fitts’s law was investigated.

### Performance comparison

The movement time shows that subjects can steer the cursor significantly faster with the non-intuitive leg control interface than the intuitive trunk control interface in relation to the 3.15 and 4.08 IDs. The sluggishness of the trunk control interface can be explained by the fact that the sEMG signals from the trunk muscles are perturbed by respiratory muscle activity and the existence of body fat tissue.

The non-intuitive control interface of the1-Dof direction performed significantly better in terms of TP and PE than the intuitive control interface. That can be explained by the fact that, despite the fact that the mechanical setup preventing any trunk or leg movement during the experiment, the subjects were still using their abdominal and back muscles to maintain an upright position. This muscle activation may have resulted in a disturbance that affected the performance. There are no significant differences in the 2-Dof due to the fact that both control interfaces needed one muscle from each pair of agonist/antagonist in order to complete the trial. There are no significant differences between the control interfaces for TP and PE but the non-intuitive control interface appears to be marginally superior.

It is notable from the path direction that the single direction targets were performed faster than the diagonal targets. This is a result of the fact that the subjects now had to activate two muscle groups simultaneously to perform the needed movement. This was significantly more difficult for the non-intuitive control mode. The poor initial performance of the intuitive control system was caused by the difficulty to activate specific muscle groups independently of each other. For example, the right and left oblique muscles are also tensed when contracting the abdominus rectus. When the subjects learnt to contract the muscles independently, they were able to control the cursor with high accuracy, resulting in a higher overall performance. The difficulty with the leg control is that it is a non-intuitive control system. When the subjects learned to associate the movement directions with the correct muscle contractions, high performance was achieved.

### Pattern recognition and abstract decoding

Several methods for control could be used except for the direct control, like pattern recognition or abstract decoding [18, 19]. The techniques of pattern recognition requires several sEMG signals, which requires more time to be attached to the skin. Additionally, due to the fact that in this approach we used extrinsic muscles, abstract decoding could lead in unpredictable training time [19].

### Future work

Even though we found in this study that the leg muscles have a better performance, the muscles of the trunk should be kept active otherwise they will be weakened even more. It would be useful then for the people with DMD to keep their muscle active through rehabilitation by playing a game with the same format as the experiment of this study. Alternatively, it is possible to combine the information of both muscles for better performance. For example, the muscles of the leg could only control the active trunk support if and only if the muscles of the trunk are activated.

## Conclusion

The non-intuitive control interface shows a tighter relation with the index of difficulty compared to the trunk and, performance is significantly higher with a higher index of difficulties. While trunk control is the more intuitive control interface, the non-intuitive leg control interface proves to be faster. Moreover, both control interfaces show similar behaviors in the learning phase. Therefore, non-intuitive control can be considered to be a viable control technique. As a result, to answer the research question, the non-intuitive control interface can be used for controlling an active trunk support. Future work will include a more elaborated analysis of both control interfaces with a higher diversity of targets, and a larger control group including people with DMD.
